# Granitic intrusions enhance strain localization and rapid mantle exhumation along an oceanic detachment fault

**DOI:** 10.1126/sciadv.aec6950

**Published:** 2026-06-03

**Authors:** Eirini M. Poulaki, Manon Bickert, Paola Vannucchi, Brandon D. Shuck, Norikatsu Akizawa, Ashutosh Pandey, Tomoaki Morishita, Alessio Sanfilippo, Emily H. Cunningham, Jaime D. Barnes, Joshua M. Garber, Claudiu Nistor, Rachel Bernard, Riccardo Tribuzio, Matthew Loocke, Noriaki Abe, Agata Di Stefano, Irina Y. Filina, Qi Fu, Swanne B. L. Gontharet, Lorna E. Kearns, Ravi Kiran Koorapati, Chao Lei, Maria Filomena Loreto, Luca Magri, Walter Menapace, Victoria L. Pavlovics, Philippe A. Pezard, Milena A. Rodriguez-Pilco, Xiangyu Zhao, Marta Pérez-Gussinyé, Carlos J. Garrido, César R. Ranero, Emily R. Estes, Alberto Malinverno, Nevio Zitellini

**Affiliations:** ^1^ Department of Geology and Geophysics, Louisiana State University, Baton Rouge, LA, USA.; ^2^ Geo-Ocean, Univ Brest, CNRS, IFREMER, UMR6538, F-29280 Plouzané, France.; ^3^ Earth Sciences Department, University of Florence, Florence, Italy.; ^4^ Earth and Planetary System Science, Hiroshima University, Hiroshima, Japan.; ^5^ School of Earth, Environmental and Sustainability Sciences, Indian Institute of Science Education and Research Thiruvananthapuram, Thiruvananthapuram, India.; ^6^ School of Geosciences and Civil Engineering, College of Science and Engineering, Kanazawa University, Kanazawa, Japan.; ^7^ Volcanoes and Earth’s Interior Research Center, Research Institute for Marine Geodynamics, Japan Agency for Marine-Earth Science and Technology, 2-15 Natsushima, Yokosuka, Kanagawa, 237-0061, Japan.; ^8^ Department of Earth and Environmental Science, University of Pavia, Pavia, Italy.; ^9^ Department of Geology and Geophysics, University of Utah, Salt Lake City, UT, USA.; ^10^ Department of Earth and Planetary Sciences, The University of Texas at Austin, Austin, TX, USA.; ^11^ Department of Geosciences, The Pennsylvania State University, University Park, PA, USA.; ^12^ School of Earth & Environmental Sciences, University of St Andrews, St Andrews, UK.; ^13^ Department of Geology, Amherst College, Amherst, MA, USA.; ^14^ Tono Geoscience Center, Japan Atomic Energy Agency (JAEA), Tokyo, Japan.; ^15^ Geological Survey of Japan, National Institute of Advanced Industrial Science and Technology (AIST), Tsukuba, Japan.; ^16^ Department of Biological, Geological and Environmental Science, Earth Science Division, University of Catania, Catania, Italy.; ^17^ Earth and Atmospheric Sciences, University of Nebraska-Lincoln, Lincoln, NE, USA.; ^18^ Department of Earth and Atmospheric Sciences, University of Houston, Houston, TX, USA.; ^19^ Laboratoire d'Océanographie et du Climat: Expérimentations et Approches Numériques, Sorbonne Université, Campus Pierre et Marie Curie, Paris, France.; ^20^ School of Earth and Environment, University of Leeds, Leeds, UK.; ^21^ Department of Earth Sciences, Binghamton University, Binghamton, NY, USA.; ^22^ College of Marine Science and Engineering, China University of Geosciences, Wuhan, China.; ^23^ Institute of Marine Sciences (ISMAR), Italian National Research Council, Rome, Italy.; ^24^ Institute for Marine and Antarctic Studies (IMAS), University of Tasmania, Hobart, Australia.; ^25^ EarthByte Group, School of Geosciences, The University of Sydney, Sydney, Australia.; ^26^ Department of Marine Geosciences, ICM-CSIC, Barcelona, Spain.; ^27^ Geosciences Montpellier, CNRS, Montpellier, France.; ^28^ Department of Marine Biology, Texas A&M University at Galveston, Galveston, TX, USA.; ^29^ School of Oceanography, Shanghai Jiao Tong University, Shanghai, China.; ^30^ MARUM–Center for Marine Environmental Sciences, University of Bremen, Bremen, Germany.; ^31^ Instituto Andaluz de Ciencias de la Tierra (IACT-CSIC), CSIC, Armilla, Granada, Spain.; ^32^ Barcelona Center for Subsurface Imaging, Institut de Ciéncies del Mar (CSIC), Barcelona, Spain.; ^33^ International Ocean Discovery Program, Texas A&M University, College Station, TX, USA.; ^34^ National Science Foundation, Alexandria, VA, USA.; ^35^ Lamont-Doherty Earth Observatory, Columbia University, Palisades, NY, USA.

## Abstract

Serpentinization and magmatism weaken the lithosphere and facilitate mantle exhumation during magma-poor rifting and ultraslow seafloor spreading. However, the complex interplay and timing among these competing mechanisms along detachment faults remain poorly constrained. The International Ocean Discovery Program (IODP) Expedition 402 drilled an incipient oceanic basin in the Tyrrhenian Sea offshore Italy. Recovered cores consist of one sequence of variably deformed granitic intrusions intercalated with slivers of peridotites and another of primarily serpentinized peridotites with heterogeneous deformation and local granitic intrusions. Geochronological and geochemical data reveal that the granites crystallized at 4 million years ago at a depth of ~7 to 9 kilometers and rapidly exhumed within ~0.5 million years, requiring exhumation rates of ~2 centimeters per year. Structural observations show that these granites accommodated significant postcrystallization strain and enhanced localization of detachment faulting. Stable isotopes record serpentinization temperatures of ~200°C, suggesting that serpentinization occurred after the emplacement and deformation of granites. We conclude that weak felsic rocks may enhance strain localization along detachment faults at intermediate depths and aid continental breakup and mantle exhumation.

## Teaser

Rheologically weak felsic intrusions enhance detachment fault nucleation, strain localization, and mantle exhumation.

## INTRODUCTION

The exhumation of deep-seated mantle rocks along divergent plate boundaries has been widely recognized at the continent-ocean transitions (COTs) of magma-poor rifted margins and along ultraslow to slow-spreading mid-ocean ridges (MORs). In these settings, the relatively low magma supply and irregular melt extraction lead to a thicker brittle layer, favoring the formation of large-scale faulting that accommodates most plate divergence. Cumulative slip along large offset normal faults results in the exhumation of the deep lithosphere to the seafloor, giving the unique opportunity to understand fault mechanics and rheological behavior, which are still poorly known (*1*, *2*). These detachment faults, which evolve to form core complexes with a distinct concave-downward fault geometry, root into a broader shear zone below the brittle-ductile transition (*3*–*7*). Previous studies have shown that sustained detachment faulting, particularly oceanic detachment faults (ODFs) that exhume large mantle sections to the seafloor, requires substantial lithospheric weakening and strain localization. Geophysical and geological observations, as well as geodynamic modeling of ODFs at both COTs and MORs (*4*, *8*–*12*), suggest that weakening is likely achieved by melt injection, mantle serpentinization, and/or rheological weakening mechanisms (e.g., grain size reduction).

At slow MORs, regular yet small-volume melt injections at and near the brittle-ductile transition are thought to decrease rock strength, thereby promoting strain localization, and long-term exhumation of mantle sections (*4*, *12*–*14*). In addition, geological and petrological observations from MOR settings reveal localized deformation at and near contact with gabbroic rocks or mafic veins, suggesting that magmatic products, which are weaker than mantle rocks, may focus strain even after crystallization (*9*, *10*, *13*, *15*–*19*). Ophiolites also show evidence of preferential melt migration through permeable fault zones locally leading to strain weakening (*20*). In addition to melt injections, alteration processes like serpentinization (i.e., hydration of olivine-rich rocks) decrease rock strength and enhance strain localization processes at lower temperatures in the upper layer of the brittle lithosphere, where fluids readily percolate via fault-enhanced permeability (*7*, *13*, *21*, *22*). Last, grain size reduction and associated deformation mechanisms (e.g., dynamic recrystallization) enhance strain localization by focusing fluid and favoring fluid-related reactions along these deformed areas (*8*, *11*, *23*, *24*). Still, the complex interplay between these various weakening mechanisms (grain size reduction, melt intrusion, and serpentinization) along the ODF is difficult to establish without robust constraints on the absolute and relative timing of these processes.

Even where exhumed mantle rocks have been sampled through dredging, in-situ sea floor sampling, and ocean drilling, the precise timing of melt injection during ODF formation in MORs and COTs has generally not been well constrained because ultramafic and mafic rocks typically have few minerals suitable for geochronology. Recent advances in for zircon geochronology and the recovery of zircon-bearing gabbros and felsic veins over the past two decades (*14*, *16*, *25*–*27*) have yielded ages that can directly date magmatic intrusions and thus constrain their relationship to tectonic extension along ODFs. Multiple geochronometers, structural characterization, and geochemical constraints are needed to establish the timing between melt injection, serpentinization, and grain-size reduction mechanisms and the feedback between them that collectively influence strain rates and detachment fault weakening. Most ophiolite analogs and samples recovered from ODFs have experienced pervasive tectonic overprinting, which makes it difficult to establish precise constraints on the temporal and spatial evolution of ODFs. Furthermore, the context of mature COT settings, where paleo–detachment faults and exhumed mantle sections are mostly inferred from geophysics data, is largely inaccessible to direct sampling, hindered by a thick sedimentary cover.

**Fig. 1.**
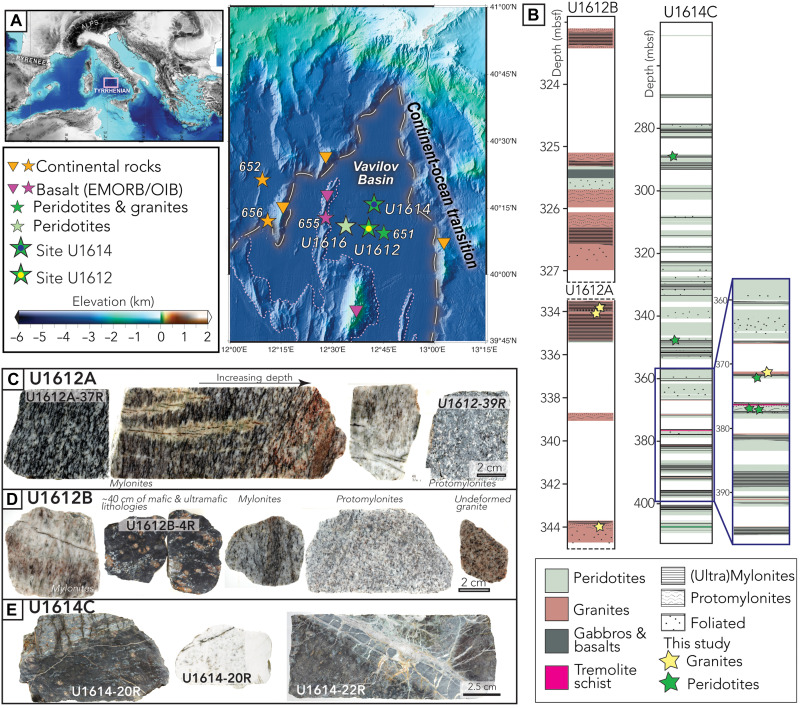
Map, recovery, and core photos. (**A**) Map of the Tyrrhenian Sea with drilled locations during Expedition 402 and ODP 107 Leg, from (*29*). Bathymetry grid is from the EMODnet portal (https://emodnet.ec.europa.eu/en/). (**B**) Core summary at Expedition 402 Sites U1612 and U1614 with lithologies, structural fabric indicators, and locations of samples used in this study. Yellow and green stars represent locations of the granites and peridotites samples used in this study, respectively. mbsf, meters below seafloor. (**C** to **E**) Representative recovered granites and surrounding mafic and ultramafic lithologies with increasing depth for holes U1612A, U1612B, and U1614C.

Here, we present the detailed analysis of in situ sections of serpentinized mantle peridotites with magmatic intrusions drilled at a young COT in the Tyrrhenian Sea during the International Ocean Discovery Program (IODP) Expedition 402 (*28*, *29*). The Tyrrhenian back-arc basin formed after Ionian lithosphere subduction beneath the Apennine chain and subsequent Calabrian slab rollback along southern Italy (*30*, *31*). Extension and crustal thinning in the Tyrrhenian Basin began in the Middle Miocene with increasing extension from north to south (*32*), ultimately leading to the opening of a V-shaped oceanic domain called the Vavilov Basin (Fig. 1) (*33*, *34*). The Vavilov Basin lies in the north-central part of the Tyrrhenian Sea where extension was briefly active between ~5 to 2 million years ago (Ma) (*31*). The locus of back-arc magmatism and extension then migrated to the southeastern Tyrrhenian Sea and formed the Marsili Basin. Extension in the Vavilov Basin ceased since the Pleistocene may be experiencing tectonic inversion at present day (*35*). Magmatism in the Tyrrhenian Basin is sparse and characterized by localized basaltic seamounts of alkaline affinity, such as the Vavilov Seamount, and basaltic ridges of tholeiitic affinity (*36*). The central Vavilov Basin, distinguished by its unique seismic velocity structure, has been interpreted by previous studies as a wide zone of exhumed serpentinized mantle peridotites (*37*, *38*). This was supported by the recovery of a 30-m-thick section of serpentinized peridotites in the Vavilov Basin during Ocean Drilling Program (ODP) Leg 107 (*34*, *39*). IODP Expedition 402, from February to April 2024, revisited the Tyrrhenian Basin and drilled four sites in the Vavilov Basin, with the goal of sampling the exhumed mantle to better understand their rheological behavior and the timescales of magmatic episodes and mantle exhumation along a COT setting (*28*).

IODP Expedition 402 penetrated through a thin veneer of overlying sedimentary cover and sampled below a heterogeneous suite of serpentinized peridotites intercalated with mafic lithologies and, notably, granites (*28*, *40*, *41*). Here, we show that the recovered felsic rocks bear well-established geochronometer minerals that offer a unique opportunity to evaluate the timing of magmatic intrusions and their relationship to deformation and serpentinization processes during mantle exhumation. We report the crystallization ages of these Tyrrhenian granites, using high-precision laser ablation split-stream inductively coupled plasma mass spectrometry (LA-SS-ICPMS) to measure U-Pb and trace element concentrations of zircon and apatite within the felsic units, coupled with microstructural observations from mylonitic to undeformed granitoids to characterize deformation mechanisms and their crystallization conditions. The zircon ages and microstructural constraints reveal that these felsic rocks are granitic intrusions emplaced during the early opening of the basin at ~4 Ma, which accommodated substantial ductile strain during exhumation of the entire heterogeneous mantle section along the detachment fault. Using multiple chronometers, textural relationships, and calcareous nannofossil ages from the overlying sediments, we show that this mantle section was rapidly exhumed at rates of ~2 cm/year over ~0.5 Ma. The total plate divergence rate during opening of the Tyrrhenian Sea is estimated to be ~2 cm/year (*30*); thus, our data suggest that most plate divergence in the basin for a brief period was accommodated by a single ODF. Oxygen and hydrogen stable isotope ratios from granite-adjacent peridotites suggest low-temperature serpentinization limited to shallow conditions. We conclude that magmatic intrusions, especially weak felsic lithologies in minor proportions, can markedly reduce lithospheric strength even after crystallization by accommodating strain at lower yield stress and, therefore, may contribute to efficient and rapid mantle exhumation along faulting-dominated COT and mature oceanic settings.

## RESULTS

### Recovery, microstructures, and deformation temperatures of granites at the Tyrrhenian COT

**Fig. 2.**
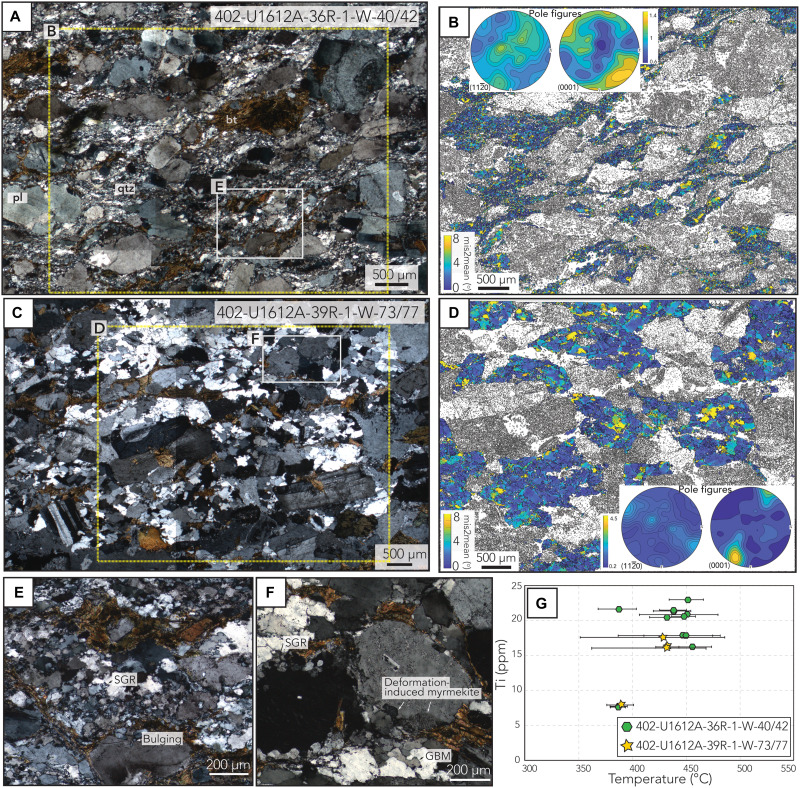
Granite microstructures, EBSD maps, and Ti-in-quartz thermometry. (**A**) Microphotograph (cross-polarized light) of mylonite in Hole U1612A, showing fine-grained recrystallized quartz accommodating deformation by dislocation creep. (**B** and **D**) Mis2mean map of quartz with up to 8° of misorientation relative to the mean orientation. Pole figure of quartz for <c> and <a> axes. Note that color bars are different for Mis2mean and pole figures. (**C**) Thin section of protomylonite (cross-polarized light) showing a coarser grain size and microstructures and GBM in quartz. (**E** and **F**) Close-up pictures highlighting deformation mechanisms observed in (A) and (C). (**G**) Ti in quartz from both samples showing similar and overlapping temperatures with those estimated from opening-angle thermometry in the mylonitic sample. qtz, quartz; Bt, biotite; Pl, plagioclase; BLG, bulging; GBM, grain boundary migration; SGR, subgrain rotation.

We focus on two IODP Expedition 402 sites that penetrated basement crystalline rocks in the central Vavilov Basin. Sites U1612 and U1614 mainly recovered heterogeneous ultramafic lithologies with some mafic intrusions, which are, overall, far more compositionally and structurally complex than samples from other COT and mature MORs (*29*). Both sites recovered felsic granitic lithologies interbedded with ultramafic and minor mafic rocks: Site U1612 (Holes A and B) recovered an ~5-m-thick massive sequence of variably deformed granitic gneisses (75% of recovered basement cores) intercalated with centimeter-thick slivers of peridotites and basalts. Just ~15 km away, Hole U1614C recovered a heterogeneous section dominated by serpentinized peridotites (*28*, *29*) with small intervals of granitic rocks forming less than 1% of the recovered basement rocks and mostly occurring in the deepest parts of the hole (Fig. 1, A and B). The co-occurrence of peridotites, mafic rocks, and felsic rocks highlight a lithological heterogeneity that, until now, has only been associated with chemically differentiated magmatic rocks along slow MORs (*13*, *14*). Silica-rich felsic veins are commonly found in minor proportions in the lower oceanic crust in MOR settings and ophiolites (*27*, *42*) and more rarely in mantle rocks from orogenic contexts (*43*). The recovered felsic lithologies from both Site U1612 and Site U1614 are typical magmatic I-type granites as shown by shipboard ICP–atomic emission spectroscopy data (*44*). The samples contain quartz, plagioclase, K-feldspar, biotite, and amphibole, with minor zircon, titanite, rutile, apatite, monazite, and allanite grains (Fig. 2), and they also contain ~75 wt % SiO_2_ and ~4 wt % Na_2_O (*28*, *40*, *41*). Although the petrogenesis of these granites is a topic of ongoing study, the recovery of such lithologies with well-established geochronometers offers a unique opportunity to link microstructures and rheological behavior to timing of magmatic emplacement and exhumation along an ODF in a COT context.

**Fig. 3.**
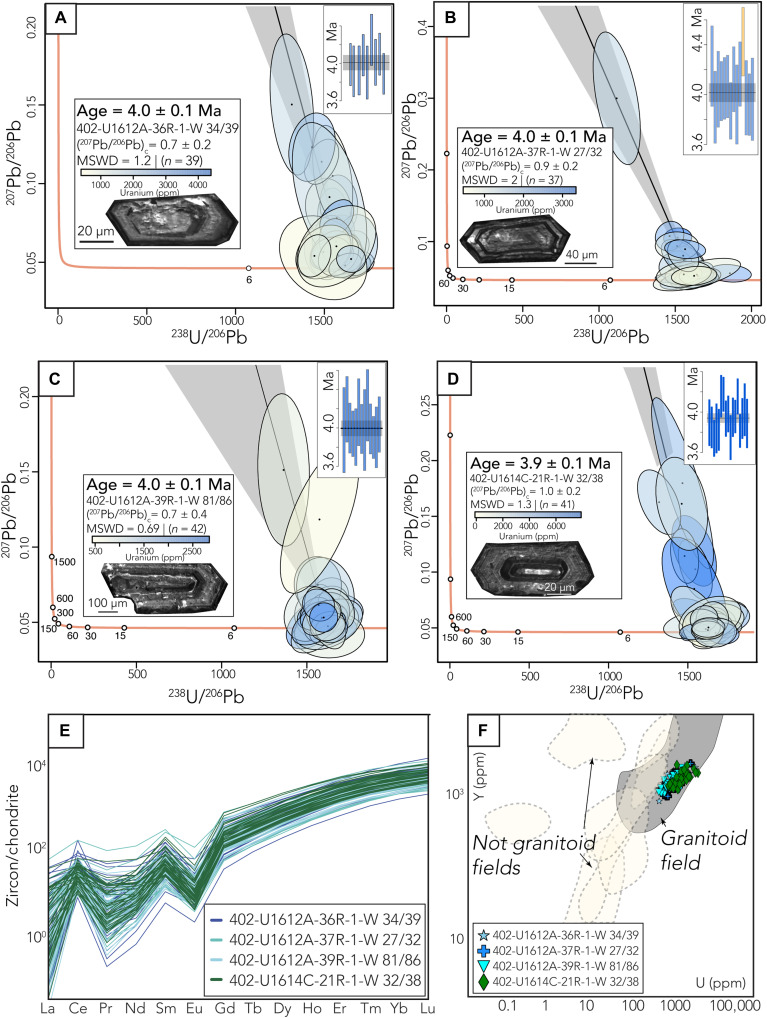
Zircon geochronologic and geochemical data. (**A** to **D**) Zircon U-Pb and trace element data from the four sampled granites in Holes U1612A and U1614C. Tera-Wasserburg plots illustrate all the analyzed zircon data (orange line is the Concordia line). Top inset: Concordia grains are plotted with the weighted mean with all ages filtered with <10% discordance. All uncertainties are 2σ errors of the mean. Bottom-left inset: CL image of the representative zircon grain from each sample and reported ages and the color bar of uranium concentrations. (**E**) Spider diagram showing trace element trends from all samples demonstrating a similar granitic composition. (**F**) Y versus U (ppm) of all the samples; discrimination field denotes continental granite zircon trace element compositions from (*47*).

In Hole U1612A, the granites show a gneissic fabric with a strong alignment of hornblende and biotite, ranging from mylonites at the top of basement to protomylonites and weakly foliated granite in the deepest parts of the hole at ~344 m below seafloor (Fig. 1B). The recovered lithologies in Hole U1612B were similar to Hole U1612A with a slight increase in the presence of intercalated ultramafic and mafic fragments (Fig. 1B). Macroscopic observations of the U1612B cores show similar decreasing mylonitization with depth and locally increasing mylonitization toward the contact with the recovered 0.4-m-thick peridotite/basalt section at the base of the hole (Fig. 1C). In Hole U1614C, core recovery was greater in sections of serpentinized peridotite but sporadic in zones of recovered granites, likely due to their friable nature or to the contrasting rheology of the two lithologies, which makes recovery more difficult. We therefore cannot exclude the possibility that thicker sections of granites exist at this site, both within the drilled section and below the base of the holes. The recovered granites in Hole U1614C, although compositionally similar to the granites from Site U1612 (*28*, *40*, *41*) (Fig. 3, E and F), only have a weak foliation fabric. However, both deformation (e.g., mylonitic peridotites with amphibole) and alteration (e.g., tremolite schist) in the surrounding mantle rocks locally increase toward the granites, which suggests a coupled system of preferential deformation and fluid circulation pathways along these lithological contrasts (Fig. 1E).

Microscopic and petrographic observations from the granitic mylonitic section (402-U1612A-36R-1W-40/42) reveal dynamic recrystallization of quartz (~30 to 50 μm in size) with subgrain boundary rotation (SGR) as the main recrystallization mechanism (Fig. 2, A and E). Large plagioclase grains (>200 μm in size) show minor deformation with undulatory extinction (Fig. 2, A and E). These observations point to deformation accommodated by dislocation creep. Quartz grains in the protomylonite sample (402-U1612A-39R-1W-73/77) are coarser (~100 to 160 μm) and dominantly characterized by minor grain boundary migration (GBM) recrystallization and SGR (Fig. 2, C and F). The coexisting plagioclase and K-feldspar in the protomylonite show undulatory extinction and minor bulging (BLG; Fig. 2, E and F). Deformation-induced myrmekite structures parallel to the foliation are also observed within plagioclase grains of the protomylonite (Fig. 2F). These structures are typically produced during upper greenschist to lower amphibolite facies conditions (i.e., 350° to 550°C) (*45*). Hornblende and biotite are oriented parallel to the main foliation fabric (Fig. 2). Electron backscatter diffraction (EBSD) data from both samples show a weak crystallographic preferred orientation (CPO) [misorientation index (MI) = ~0.1 to 0.2; see Materials and Methods] with a dominant prism and rhomb <a> slip system, indicating deformation under simple shear (Fig. 2, B and D) (*46*). Opening-angle thermometry in quartz from the mylonite (402-U1612A-36R-1W-40/42) yields deformation temperatures of ~440°C, which is comparable to temperatures of ~370° to 450°C estimated from Ti-in-quartz thermometry from both the same mylonite sample and a protomylonite sample (Fig. 2E; Materials and Methods). Because Ti diffusion in quartz is sensitive to deformation accommodated by dislocation creep, we interpret these temperatures as representing the temperatures of quartz recrystallization during ductile deformation in the ODF shear zone at moderate depths.

The observed grain size reduction and transition in dynamic recrystallization mechanisms from quartz SGR (mylonite) to GBM (protomylonite), together with the similar deformation temperatures (~370° to 450°C) between the two samples, suggest that the primary difference in microstructures between the mylonite and protomylonite is related to differences in the amount of accommodated strain (*46*). Together, our macro- and microstructural observations argue that the granites intruded into an incipient ODF shear zone and subsequently accommodated postcrystallization strain via compositional weakening that facilitated mantle exhumation.

### Timing and emplacement conditions of the granites

All analyzed granitic samples (table S1) from Hole U1612A yield indistinguishable and overlapping zircon crystallization ages of 4.0 ± 0.1 Ma (Fig. 3, A to C; Materials and Methods). Similarly, a granitic sample from Hole U1614C yields a zircon crystallization age of 3.9 ± 0.1 Ma, overlapping within error with ages estimated for U1612A (Fig. 3D). The zircon trace element signatures from all samples are similar and display elevated Heavy Rare Earth Elements (HREE) in comparison to Light Rare Earth Elements (LREE) and negative Eu and positive Ce anomalies (Fig. 3F), which is consistent with crystallization from a felsic magma in continental settings (*47*). Cathodoluminescence (CL) images from zircon grains show euhedral crystals with oscillatory zoning, confirming their magmatic origin (Fig. 3, A to D). Zircon has been shown to undergo rim growth during hydrothermal alteration at temperatures as low as 350°C (*48*). Here, despite hydrothermal alteration in the surrounding lithologies, we do not find any geochemical evidence of postcrystallization alteration of the zircon grains. Ti in zircon and Ti in hornblende temperature calculations yield similar estimates of zircon and granite crystallization temperatures of 630° ± 30°C and ~700° ± 45°C, respectively (Materials and Methods). In addition, we estimate crystallization pressures of ~0.2 GPa from Al in hornblende geobarometry (Materials and Methods). Using these temperature and pressure estimates, we calculated granite crystallization at a depth of ~7 to 7.5 km considering an average overburden rock density of 2.7 to 2.9 g/cm^3^ derived from shipboard bulk density measurements (*41*).

**Fig. 4.**
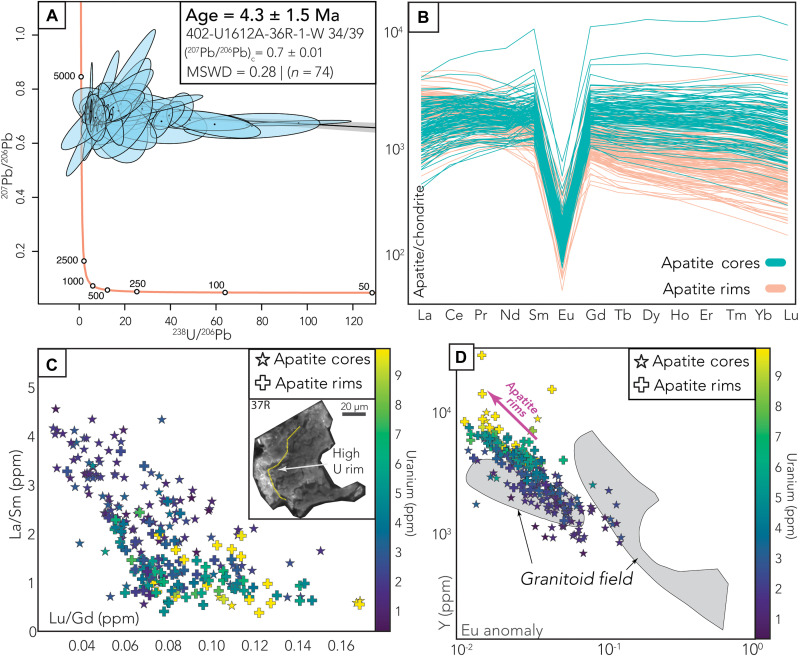
Apatite geochronologic and geochemical data. (**A**) The Tera-Wasserburg plot of apatite grains from the sample 402-U1612-36R-1-W-34/39 yields an age of 4.3 ± 1.5 Ma. (**B**) Apatite trace element spider diagrams illustrating differences in the geochemical composition of apatite cores versus rims. Cores have enriched HREE and a steeper LREE slope in comparison to the apatite rims. (**C**) Apatite trace element data of La/Sm versus Lu/Gd and are colored on the basis of the uranium concentration of each grain and grouped on the basis of rim versus core. (**D**) Y (ppm) versus Eu anomaly of all the samples grouped by cores and rims; discrimination field denotes granitoid apatite trace element compositions from (*49*). Note that rims do not plot in the granitoid field and have increased Y and U.

Apatite grains from only one mylonitic sample (402-U1612A-36R-1W-34/39) yielded comparable U-Pb ages of 4.3 ± 1.5 Ma, which overlap within error with the zircon ages, despite a larger uncertainty (Fig. 4A). The other apatite-bearing samples yield large errors due to high common Pb and low U concentrations (Fig. 4A), with most of them preserving high U concentrations at the rim and substantially different trace element concentrations compared to their cores (Fig. 4B). The apatite cores have trace element patterns with mostly flat HREE, a gentle negative LREE slope, and a negative Eu anomaly. We interpret the trace element patterns and the U-Pb ages of 4.3 ± 1.5 Ma of apatite cores as a magmatic signature (*49*) that formed at the same time as zircon during crystallization of the granites.

In contrast, although they did not yield a distinguishable age, the U-rich apatite rims with higher Y and depleted HREE in comparison to the cores suggest a postcrystallization alteration of hydrothermal origin (Fig. 4, B to D) (*50*). These high U concentration rims have irregular boundaries, a patchy texture, and high luminescence that is clear in the CL images (Fig. 4C). Apatite grains are susceptible to hydrothermal fluids and can dissolve and reprecipitate, recording these changes in their trace element concentrations (*51*). Therefore, we propose that apatite rim formation likely postdates the emplacement of the granites and is associated with a low-temperature hydrothermal alteration event, likely reflecting the onset of seawater infiltration into the ODF shear zone.

### Nature of serpentinization

**Fig. 5.**
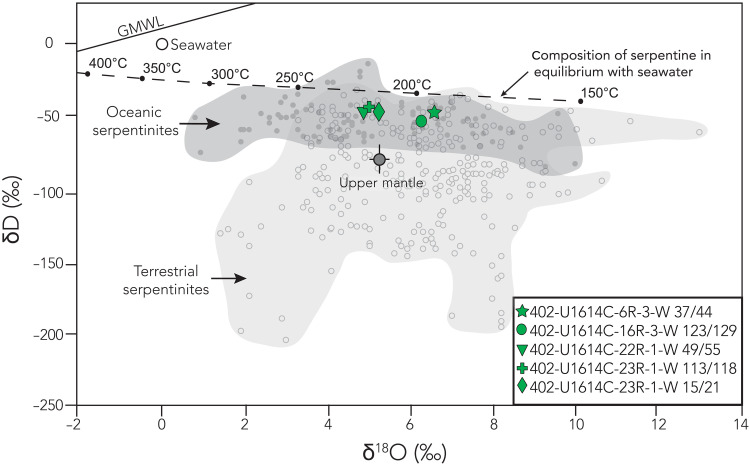
Oxygen and hydrogen isotope analyses. Oxygen and hydrogen isotope compositions of serpentinized peridotite from Hole U1614C. The dashed black line shows the isotopic composition of serpentine in equilibrium with seawater (0‰) at various temperatures using the oxygen and hydrogen isotope fractionation factors of (*53*). The isotopic compositions of seafloor (dredged or drilled) serpentinites and terrestrial serpentinites are shown for reference, as well as the average composition of the upper mantle. GMWL, global meteoric water line. Figure modified from (*106*), with references for data compilation therein.

To estimate the fluid sources and temperature range of mantle serpentinization, we separated inclusion-free serpentine from the matrix of U1614 peridotite samples to obtain oxygen isotope ratios and excluded any crosscutting serpentine veins that may correspond to later and cooler serpentinization stages (Materials and Methods). Serpentine δD and δ^18^O values from the serpentinized peridotite matrix range from −44 to −54 per mil (‰) and +3.8 to +6.3‰, respectively (Fig. 5), consistent with published data from oceanic abyssal serpentinites (*52*). The high δD values argue for serpentinization via seawater with limited input of magmatic fluids. Using the oxygen isotope serpentine-water fractionation factor of (*53*), we estimate temperatures of serpentinization at ~200° to 225°C (Fig. 5 and table S4; Materials and Methods). The calculated serpentinization temperature is ~450°C cooler than our estimated temperatures for granite emplacement temperature and ~250°C cooler than those for ductile deformation temperatures. This suggests a clear evolution from melt crystallization at depth, to ductile strain during early exhumation, and finally late-stage serpentinization at shallow depths. Previous microstructural and oxygen isotope work on serpentinites from the Iberian COT and from slow MOR settings have found similar low temperatures of serpentinization and interpreted this as resulting from seawater-derived fluids percolating along an existing detachment fault (*22*, *54*, *55*). The small temperature range in stable isotope data suggests a low-temperature primary serpentinization event that pervasively altered the ultramafic section exhumed in the Vavilov Basin of the Tyrrhenian Sea.

## DISCUSSION

### Granites enhance strain localization during exhumation

Our macro- and microstructural data from deep-sea drilling of an in situ heterogeneous mantle section along the Tyrrhenian COT show that the granites spatially coincide with a local increase in deformation intensity. Coupled geochronometers and mineral thermometry and barometry with microstructures clearly demonstrate that the granitic melts intruded into the mantle at the onset of basin oceanization and then subsequently experienced ductile deformation that postdates their magmatic crystallization (Fig. 2). At the recorded granite deformation temperatures of ~400° to 450°C, the lower yield strength of quartz and feldspar resulted in granite viscous deformation at lower stresses, whereas olivine, and hence the surrounding unaltered peridotites, is relatively stronger and has already passed through their brittle-ductile transition at higher temperatures (~750°C) (*46*, *56*, *57*). The emplaced granites, weaker than the primarily dry olivine in the host mantle peridotites, favor strain localization along these newly formed compositional and rheological heterogeneities, evidenced by grain size reduction and localized dislocation creep in the felsic material near the contact with ultramafic lithologies. In turn, these deformed zones weaken the host rock section, further focusing and enhancing strain and fluid/melt circulation pathways. Comparable weakening mechanisms have been observed in oceanic transform and detachment faults, in which the addition of fluids or melts led to grain size reduction, with local activation of dissolution-precipitation creep (*4*, *9*, *24*, *58*, *59*). Plagioclase in gabbros from MORs has also been shown to deform by dislocation creep especially in plagioclase-rich mylonitic layers (*60*). Our results further support that even minor proportions of chemically fractionated magmatic rocks can help stabilize detachment faulting through local compositional weakening, strain focusing, and an overall decrease in the bulk strength of the shear zone.

Despite the presence of refertilized lherzolitic mantle, dunite channels, and mafic and felsic intrusions (*29*) and the recovery of basalts in ODP Hole 651 (*34*), IODP Expedition 402 found no evidence for the presence of continuous, normal oceanic crust in the Tyrrhenian Sea. Instead, the Vavilov Basin is largely dominated by a heterogeneous section of exhumed mantle that experienced episodes of melt infiltration and hydrothermal alteration. It is likely that mantle melts were too volumetrically minor to form robust oceanic crust and, instead, these melts exploited preexisting lithological heterogeneities observed in the Tyrrhenian basement rocks (e.g., dunite channels and refertilized lherzolite) (*29*), which agrees with previous findings in slow MOR settings that show connections between strain localization and rheological heterogeneities such as gabbro/peridotite contacts (*13*, *15*, *61*). Therefore, we propose that detachment faulting in the Tyrrhenian Sea was enhanced by magmatic processes that contributed to strain localization and facilitated widespread mantle exhumation. Our results are also in good agreement with prior petrological studies of detachment faults along MORs, ophiolite complexes, and numerical models (*11*, *12*, *20*), which argue that shear zones promote melt infiltration and local thermal weakening.

### Constraints on exhumation rates along an ODF

By coupling different chronometers and geochemical analyses in the recovered granites, we estimated the minimum exhumation rates of the mantle section by the detachment fault. Zircon U-Pb geochronologic data from the granites yield zircon crystallization ages of 3.9 to 4.0 Ma with ± 0.1 Ma uncertainty. To determine the depth of the granite emplacement, we used the Al in amphibole geobarometer, which indicates pressures of 0.2 to 0.3 GPa (Materials and Methods and table S5). This pressure corresponds to depths of ~7 to 9 km, using an average overburden lithospheric density of ~2.7 to 2.9 g/cm^3^ derived from shipboard bulk density measurements (*48*). Because this basement section was recovered at 330 m below the seafloor, these constraints provide a total exhumation path of 6.7 to 8.7 km. The deepest sediments directly overlying the basement at Hole U1612A belong to the Upper Pliocene nannofossil biozone MNN15b, dating them to 2.82 to 3.56 Ma (*28*). The sediment ages constrain the minimum timing of exhumation to the seafloor because we cannot rule out a hiatus in sedimentation or unconformity at the sediment-basement contact. Therefore, the U-Pb and nannofossil age constraints provide a maximum duration of 0.44 million years (Myr) from emplacement of the granites at depth to their complete exhumation at the seafloor. Across the detachment fault at Hole 651A, a reexamination of the biostratigraphy and chronology of the sediments overlying the basement (*62*) yields a compatible age of 4.1 to 4.2 Ma. The comparable basal sediment ages overlying the basement are slightly younger than or similar to ages derived from zircon geochronology, suggesting that the granitic intrusions were emplaced shortly before, or coeval with, detachment fault nucleation at ~4 Ma. Seismicity and morphological observations from ODFs suggest a dip angle ranging from ~50° to 70° at greater depths to ~20° at shallow depths (*63*, *64*). Considering a conservative angle of 60°, our multichronometer constraints imply a minimum exhumation rate of ~1.85 to 2.27 cm/year; hence, we report an average of ~2 cm/year. This rate is remarkably similar to basin-scale extension rates of 2 cm/year estimated for the opening of the Tyrrhenian Sea (*30*). Our results suggest that between ~4 and 3.5 Ma, regional extension across the COT in the Vavilov Basin was perhaps accommodated by a single, deeply rooted ODF.

Our estimated exhumation rates are similar to slightly higher than those estimated along slow and ultraslow MORs. For instance, studies on the Mid-Atlantic Ridge and the nearly amagmatic Eastern Southwest Indian Ridge calculated spreading rates of 1 to 1.4 cm/year, where ODFs accommodate up to 50 to 80% of total plate divergence (*61*, *65*). At Atlantis Massif and Atlantis Bank, exhumation rates were determined to be 1.4 to 2 cm/year, which corresponds to ~80% of the full spreading rate in the Antarctic plate and ~70 to 100% in the North American plate, respectively (*14*, *66*). Similarly, our calculated ODF exhumation rate of ~2 cm/year in the Tyrrhenian Sea corresponds to the inferred plate divergence rate, requiring a high degree of strain localization along the detachment fault. This suggests that the granites and gabbros similarly provide a weaker rheology than surrounding dry peridotites and contribute to strain localization by lubricating detachment faults to stabilizing long-term slip and exhumation of mantle rocks. The main difference would likely be due to the range of temperatures at which granite (predominantly quartz and plagioclase) and gabbro (plagioclase/pyroxene/olivine) cross their respective brittle-ductile transitions. Gabbroic intrusions would deform ductilely at temperatures as low as ~550°C with most studies reporting brittle-ductile transitions of gabbro in oceanic settings of >650°C (*9*, *67*). In contrast, felsic rocks with quartz and feldspar can continue deforming ductilely at temperatures as low as ~350°C. Thus, low-viscosity granites may accelerate deformation and promote more efficient and rapid strain localization in moderate structural depths of the detachment fault.

### The secondary role of serpentinization in detachment fault nucleation

With the in situ recovery of these granitic mylonites along with serpentinized peridotites, we observe a fossil incipient shear zone with a clear relationship between increasing mylonitization and proximity to the serpentinized peridotites. Several lines of evidence from the chemical, thermal, and mechanical footprint of the exhumed record suggest that serpentinization occurred after the observed ductile deformation in the granites. Trace elements from the apatite cores (Fig. 4B) and zircon grains (Fig. 3F) show that they formed coevally during early crystallization, whereas the presence of postcrystallization apatite rims and the absence of zircon rims suggest that hydrothermal alteration occurred at lower temperatures. Additional evidence of late-stage serpentinization comes from the textural observations of alteration products. The process of serpentinization is known to generate hyperalkaline, Ca^2+^-rich fluids that metasomatize adjacent silicate rocks into calc-silicate assemblages (e.g., rodingites) and/or talc-rich mixed lithologies (*68*, *69*). Mesoscopic observations from Hole U1614C reveal the presence of mostly undeformed carbonate-bearing and calc-silicate–bearing veins crosscutting the peridotite fabric (*28*) or folded talc schist. The latter is much weaker than granite and probably deformed after the granites had crossed their brittle/ductile transition. This textural relationship indicates that the metasomatic event—and by direct extension, the serpentinization—must have occurred after the cessation of the high-temperature (<430°C) ductile deformation recorded in the granites.

The thermal state of the host mantle at the time of granite emplacement also provides a critical constraint on the relative timing of serpentinization. If the granitic magma, which crystallized at temperatures of ~630° to 700°C based on amphibole thermobarometry (Materials and Methods), intruded into a preexisting, cooler (~200° to 250°C) and hydrated serpentinized peridotite, the resulting thermal gradient would have produced a distinct contact metamorphic aureole. Natural analogs, such as the Bergell granite intrusion in the Malenco ophiolite, demonstrate that such aureoles are characterized by a sequence of prograde dehydration reactions, forming isograds marked by the appearance of talc, tremolite, and metamorphic olivine and orthopyroxene (*70*, *71*). The absence of such high-temperature contact metamorphic aureole in the IODP 402 cores is compelling evidence against the presence of substantial serpentinization at the time of melt intrusion. Collectively, these points argue that the granites were emplaced into largely anhydrous lithospheric peridotite.

Last, the mechanical behavior of the recovered lithologies provides a final, decisive constraint on the relative timing of serpentinization and its relationship with the deformation of the granites. Our microstructural data show that the granites localized strain at ~450°C (Fig. 2G) with deformation accommodated by dislocation creep (Fig. 2, A to F). At these temperatures, the stable serpentine polymorph, antigorite, is known from experimental studies to be exceptionally weak, with a flow stress at least an order of magnitude lower than that of wet quartzite, a common analog for granite rheology (*72*, *73*). A previous study in the Tyrrhenian Sea using samples from ODP Leg 107 showed that most serpentine polymorphs are chrysotile and lizardite and noted the absence of antigorite (*39*). Besides, if large volumes of antigorite had been present, the strong rheological contrast would have forced strain to partition almost exclusively into the serpentinite. This predicted structural style is in direct contradiction to our core observations, which show mylonitized granites. Hence, the low serpentinization temperatures (~230°C; Fig. 5), and the observations of serpentinized veins preferentially along preexisting rheological heterogeneities described in (*29*), suggest that serpentinization primarily occurred after the onset of deformation and partial unroofing of this mantle section. Coeval local serpentinization at the shallow portion of the fault is still plausible at the time; however, serpentinization did not penetrate to greater depths where our sampled section of granites and mantle rocks were deforming under relatively higher-temperature conditions.

### Competing weakening mechanisms in ODFs, from melt intrusions to serpentinization

**Fig. 6.**
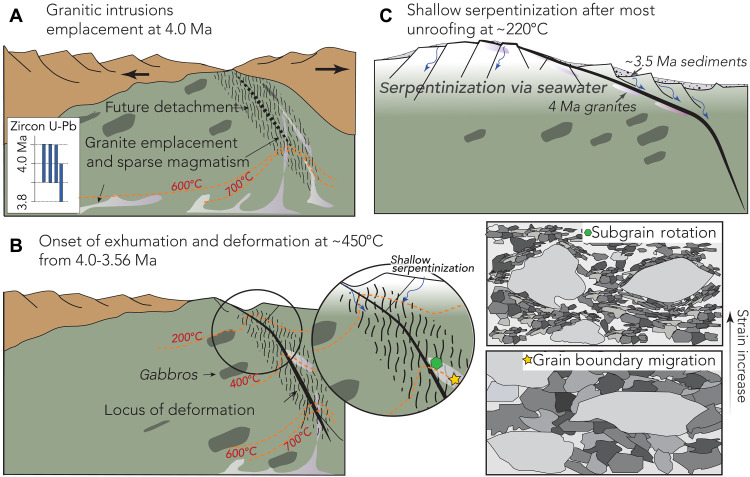
Emplacement of granites enhance detachment fault nucleation, strain localization, and mantle exhumation. Conceptual model of the interaction of granitic intrusions with an incipient ODF in the Tyrrhenian Sea based on the observations from this study and integrated with prior studies of the basin evolution. (**A**) Emplacement of granites constrained by zircon U-Pb ages and geochemical data support a syntectonic melt injection coeval with late-stage extension in the basin. We speculate that granitic melts were focused under the rift axis where active plate divergence created long-offset normal faults. (**B**) The detachment fault shear zone develops into the mantle due to strain localization along granitic intrusions, supported by EBSD data in quartz and moderate-temperature hydrothermal alteration supported by apatite trace element data. The lower-temperature brittle-ductile transition of the granites allowed them to deform in a weak viscous manner and facilitate strain localization and sustained detachment faulting at moderate depths. (**C**) Extension continues along the detachment, where shallow normal faulting and seawater infiltration causes pervasive mantle serpentinization after most unroofing. The comparatively weak rheology of serpentinized mantle favors rotation of the detachment to low angles at shallow depths, promoting further extension and forming an oceanic core complex on the seafloor.

Serpentinization is often inferred to be key in focusing extensional deformation at COTs (*7*). In contrast, our results here show that serpentinization was a secondary weakening mechanism during initial mantle exhumation at the Tyrrhenian COT. These observations are based on the lower temperatures of serpentinization (~230°C) and strain localization in the granites at higher temperatures (~450°C). Serpentinization is known to reduce rock strength, promoting brittle failure at shallower depth and low-temperature conditions during the late stages of exhumation, enabling the flexure of the footwall and exhumation of mantle rocks to the seafloor (Fig. 6C). The persistence of localized strain at the COT detachment is similar to deformation at modern ultraslow and slow-spreading MORs with moderate to low melt supply, where divergence is accommodated by both magmatic and tectonic processes and where hydration reactions control strain localization in the upper brittle lithosphere (*8*, *12*, *21*, *22*). Here, we show that magma, even in small volumes, may be effective for localization of strain at magma-poor rifted margins as the postcrystallization rheological behavior of differentiated magmatic bodies may be an overlooked compositional weakening mechanism to aid detachment faulting and mantle exhumation during the final stages of rifting. We conclude that granitic intrusions into the mantle section at the COT of the Tyrrhenian backarc basin formed a weaker felsic rheology than the surrounding mantle lithosphere, which enhanced shear deformation localization and accelerated the detachment fault activity. Hence, this fault motion might absorb most of the plate boundary extension, enabling highly localized and efficient mantle exhumation (Fig. 6). Shallow depth alteration and metasomatism, including serpentinization, continue to localize strain as the detachment is still active and through the final exhumation stages. This is also evidenced by the observed alteration minerals in the granites with chlorite and epidote/alanite alteration from core to rim.

In summary, although synkinematic mylonitic granites have not been previously reported within exhumed COT and oceanic mantle sections, our results show that weakening mechanisms within detachment faults in the Tyrrhenian Sea are not fundamentally different from those observed in other slow and ultraslow MOR settings. In the Tyrrhenian Sea, the detachment fault exhibits fast exhumation rates and strain continues to localize and facilitate exhumation under cooler temperatures, yet the underlying processes remain consistent with other more common magmatic intrusions such as gabbros. The presence of granites in IODP Expedition 402 samples offers a unique opportunity to give absolute and relative time constraints on detachment faulting processes from depths above the brittle-ductile transition of typical mafic and ultramafic rocks. These depths previously lacked many constraints due to the mafic to ultramafic nature of the recovered lithologies, including crystallization depths, strain localization, hydrothermal fluid metasomatism, and both absolute and relative timing constraints. Although the granites recovered during Expedition 402 may be scarce or seem atypical, the compositional weakening mechanisms they capture are broadly applicable to divergent plate boundary contexts, operative at shallower depths than previously observed while providing a more efficient mechanism for detachment fault lubrication and accelerating the exhumation of deep-seated mantle rocks to the seafloor.

## MATERIALS AND METHODS

### Petrographic, microstructural, and EBSD analysis

Rock billets were cut for the production of thin sections parallel to the lineation and perpendicular to the foliation. The foliation is subhorizontal throughout U1612 cores. The structure and petrology teams during IODP Expedition 402 on the *JOIDES Resolution* denoted the cutting lines of the drilled cores, and the laboratory technicians on the *JOIDES Resolution* cut the billets.

Scanning electron microscopy (SEM) work was conducted at the Molecular Analysis Facility, a National Nanotechnology Coordinated Infrastructure (NNCI) site at the University of Washington, supported in part by the NSF (NNCI-2025489 and NNCI-1542101), the Molecular Engineering & Sciences Institute, and the Clean Energy Institute. Thin sections were polished to 0.05-μm colloidal with a vibratory polisher and then coated with a 5-nm-thick carbon coat. EBSD analyses were collected using an Oxford Symmetry detector and Oxford AzTec software. Data were collected at a working distance of 12.6 mm and 70° tilt in high vacuum. Beam energy was set at 20 kV, a current of 3.2 nA, and a step size of 5 μm. The MTEX software package in Matlab was used to clean the raw EBSD data by removing spikes and noise (*74*). The cleaned EBSD data were plotted with MTEX to produce misorientation maps, pole figures, and calculate the MI, which quantifies fabric strength in deformed materials (*75*). EBSD data were also used to calculate temperatures using opening-angle thermometry, implementing the script from (*76*) and following the calculation parameters by (*77*).

### Zircon and apatite LA-SS U-Pb and trace element analyses

LA-SS depth profiling of zircon crystals was performed at the LionChron facility at Penn State. The zircon crystals were mounted on double-sided sticky tape for analysis. U-Pb isotopes and trace element data were collected simultaneously from the same spots. Samples were ablated using a Teledyne/Photon Machines Analyte G2 excimer laser ablation system with a Helex2 ablation cell, coupled to a Thermo Fisher Scientific Element XR ICPMS system for U-Pb isotopes and a Thermo Fisher Scientific iCAP-RQ ICPMS system for trace elements. All elements on both ICPMS systems were measured on secondary electron multipliers. The total Ar gas flow for the experiment was ~2.4 liter/min, with total He gas flows from the laser at 0.44 liter/min. All samples were run during the same session, and all zircons were run with a 40-μm spot size, 6-Hz repetition rate, 240 shots, and a laser fluence at the sample surface of ~5 J/cm^2^, yielding pit depths on the order of ~20 μm. The laser was first fired thrice with the same spot size to remove surface contamination, and this material was allowed to wash out for 20 s. Measured peaks on the Element XR were ^204^Pb, ^206^Pb, ^207^Pb, ^208^Pb, ^232^Th, and ^238^U. Analyses of unknowns were bracketed by analyses of matrix-matched zircon reference material (RM) 91500 [1062.4 ± 0.4 Ma ID-TIMS ^206^Pb/^238^U date (*78*)], which was used as the primary RM for U-Pb isotopic analyses. Zircon RMs GJ-1 [601.7 ± 1.3 Ma ID-TIMS ^206^Pb/^238^U date (*79*, *80*)], Plešovice [337.13 ± 0.37 Ma ID-TIMS ^206^Pb/^238^U date (*81*)], and Piexe [564 ± 4 Ma ID-TIMS ^206^Pb/^238^U date (*82*)] were used as secondary RMs, and NIST SRM 612 glass (*83*) was run as the primary trace element RM. Using the same parameters and methods applied to unknowns, we obtained U-Pb concordia dates of 596.6 ± 2.1 Ma for GJ-1, 339.8 ± 1.3 Ma for Plešovice, and 567.1 ± 2.5 Ma for Peixe during the zircon analytical session, which are accurate to 0.8, 0.8, and 0.5% of their reference values, respectively. For trace element analyses, ^29^Si (assuming 15 wt % Si) was used as an internal standard, with measured peaks on the iCAP-RQ at ^27^Al,^29^Si, ^31^P, ^49^Ti, ^89^Y, ^93^Nb, ^139^La, ^140^Ce, ^141^Pr, ^146^Nd, ^147^Sm, ^153^Eu, ^157^Gd, ^159^Tb, ^163^Dy, ^165^Ho, ^166^Er, ^169^Tm, ^172^Yb, ^175^Lu, and ^180^Hf. Iolite version 4 (*84*) was used to correct measured isotopic ratios and elemental intensities for baselines, time-dependent laser-induced inter-element fractionation, plasma-induced fractionation, and instrumental drift. The mean and SE of the measured ratios of the backgrounds and peaks were calculated after rejection of outliers more than 2 SEs beyond the mean. Downhole fractionation was modeled using an exponential best fit. We added an additional 2% error to each ^207^Pb/^235^U and ^206^Pb/^238^U ratio in quadrature to account for variation in ablation or transport characteristics, mass-balance instabilities, or plasma loading effects, which yields a single age population [mean square weighted deviation (MSWD) ≤ 1] for each of the secondary RMs. Stated 2σ date uncertainties are internal—that is, they include in-run errors only—whereas the long-term external uncertainty for zircon U-Pb in this laboratory is ~2%. We also used the Ti in zircon thermometer by (*85*). We corrected the Ti concentrations in the unknown samples based on the GJ1 Ti concentrations and reduced them by 30% to match the known Ti concentrations following the calculations after Ferry and Watson (*86*). IsoplotR (*87*) was used for all age and concordia calculations and plots. Trace element normalization and data plotting were performed using a Python code. The full zircon dataset is contained in table S2.

LA-SS depth profiling of apatite crystals was performed in the same facility, using the same instrumental setup and gas flows. The apatite crystals were mounted in double-sided sticky tape for analysis. All samples were run during the same session, and all apatites were run with a 40-μm spot size, 8-Hz repetition rate, 200 shots, and a laser fluence at the sample surface of ~5 J/cm^2^, yielding pit depths on the order of ~20 μm. The laser was first fired thrice with the same spot size to remove surface contamination, and this material was allowed to wash out for 15 s. Measured peaks on the Element XR were ^204^Pb, ^206^Pb, ^207^Pb, ^208^Pb, ^232^Th, and ^238^U. Analyses of unknowns were bracketed by analyses of matrix-matched apatite RM MAD-UCSB [467 ± 9 Ma ID-TIMS ^207^Pb/^206^Pb-^238^U/^206^Pb isochron age (*88*)], which was used as the primary RM for U-Th-Pb isotopic analyses. Apatite RMs BRZ-1 [2078 ± 12 Ma ID-TIMS ^207^Pb/^206^Pb-^238^U/^206^Pb isochron age (*88*)], MRC-1 [153.4 ± 0.4 Ma ID-TIMS ^207^Pb/^206^Pb-^238^U/^206^Pb isochron age (*88*)], Durango [31.44 ± 0.18 Ma ^40^Ar/^39^Ar reference age (*89*)], Emerald Lake [92.2 ± 0.9 Ma ID-TIMS ^206^Pb/^238^U titanite age (*90*, *91*)], and MAD2 [474.3 ± 0.4 Ma ID-TIMS weighted mean ^206^Pb/^238^U age (*92*)] were used as secondary RMs, and NIST SRM 612 glass (*83*) was run as the primary trace element RM. Using the same parameters and methods applied to unknowns, and using the published (BRZ-1 and MRC-1) or (*93*) modeled (Durango, Emerald Lake, and MAD2) upper ^207^Pb/^206^Pb intercept for each RM, we obtained ^207^Pb/^206^Pb-^238^U/^206^Pb isochron dates of 2041 ± 14 Ma for BRZ-1, 151.5 ± 1.3 Ma for MRC-1, 30.9 ± 0.7 Ma for Durango, 91.5 ± 5.9 Ma for Emerald Lake, and 471.1 ± 2.4 Ma for MAD2 during the apatite analytical session, which are accurate to 1.8, 1.2, 1.7, 0.8, and 0.7% of their reference values, respectively. For trace element analyses, ^43^Ca (assuming 39 wt % Si) was used as an internal standard, with measured peaks on the iCAP-RQ at ^7^Li, ^24^Mg, ^27^Al, ^29^Si, ^31^P, ^43^Ca, ^45^Sc, ^49^Ti, ^51^V, ^55^Mn, ^57^Fe, ^88^Sr, ^89^Y, ^90^Zr, ^93^Nb, ^139^La, ^140^Ce, ^141^Pr, ^146^Nd, ^147^Sm, ^153^Eu, ^157^Gd, ^159^Tb, ^163^Dy, ^165^Ho, ^166^Er, ^169^Tm, ^172^Yb, and ^175^Lu. The accuracy of the trace element measurements was assessed by comparing standard results to those from the same crystals reported in (*94*), which were found to be within error. Iolite version 4 (*84*) was used to correct measured isotopic ratios and elemental intensities for baselines, time-dependent laser-induced inter-element fractionation, plasma-induced fractionation, and instrumental drift. Rims and cores were selected in the Iolite software following procedures after (*95*). The mean and SE of the measured ratios of the backgrounds and peaks were calculated after rejection of outliers more than 2 SEs beyond the mean. Downhole fractionation was modeled using an exponential best fit. We added an additional 2% error to each ^207^Pb/^235^U and ^206^Pb/^238^U ratio in quadrature to account for variation in ablation or transport characteristics, mass-balance instabilities, or plasma loading effects, which yields a single age population (MSWD ≤ 1) for each of the secondary RMs. Stated 2σ date uncertainties are internal—that is, they include in-run errors only—whereas the long-term external uncertainty for apatite U-Pb in this laboratory is ~2% (*94*). IsoplotR (*87*) was used for all age and Concordia calculations and plots. Trace element normalization and data plotting and normalization to chondrite were performed by using an in-house Python code. The full apatite dataset is contained in table S3.

### Oxygen and hydrogen isotope analyses

δ^18^O values of serpentine separates and δD values of bulk serpentinites were measured at the University of Texas at Austin using a Thermo Fisher Scientific MAT 253 isotope ratio mass spectrometer. Serpentinites were prepared for δ^18^O analysis by picking ~2.0 mg of inclusion-free serpentine grains from coarsely crushed samples for measurement using the laser fluorination method of (*96*). Bulk serpentinites were prepared for δD analysis by packing ~1.5 mg of the powdered sample into silver capsules for pyrolysis in a high-temperature conversion elemental analyzer (TC-EA), following the method of (*97*). δ^18^O and δD values are reported relative to standard mean ocean water (SMOW) with errors of ±0.2‰ (1 SD) and ±2.0‰ (1 SD), respectively. Calculated temperatures of serpentinization used in the text use the fractionation factor of (*52*), which yield slightly higher temperatures than fractionation factors of other known thermometer calibrations (table S4).

### Microprobe techniques

Electron probe microanalysis data were collected using the JEOL JXA-8230 Electron Probe Microanalyzer (EPMA) in the Chevron Geomaterials Characterization Laboratory in the Department of Geology & Geophysics at Louisiana State University. CL images were collected using a JEOL panchromatic CL detector with a beam accelerating potential of 15 kV, a current of 40 nA, and a dwell time of 30 μs/pixel.

Ti-in-quartz analyses followed a routine similar to that of (*98*). Standardization was carried out on rutile and quartz, and the QTiP series of single crystals (*99*) was used as an RM. Quartz and rutile standardization was carried out using a beam current of 20 nA so as to avoid the peak shifts, which are known to be caused by the pulse-height analyzer at higher count rates (*99*). Quantitative analyses were carried out using a beam current of 200 nA and an accelerating potential of 15 kV with a focused beam. The Ti Kα x-rays were simultaneously counted on two spectrometers (total: 500-s peak and 200 s background) using an exponential background interpolation fitted to a spectrometer wavescan of a QTiP standard sample. For the analyses, Si and O were not analyzed and assumed to be stoichiometric. A Herkimer quartz standard provided by W. Nachlas and known to contain 4 to 5 ± 2 parts per billion (ppb) (*100*) was used as a blank to evaluate the background signal of the spectrometers (*101*, *102*). Our method yielded a calculated detection limit of 7 parts per million (ppm), an SE of 9 ppm, and a reproducibility of 6 to 26 ppm on analysis of QTiP standard materials. All the data can be found in table S6.

Amphibole analyses (table S5) were collected using an accelerating potential of 15 kV, a beam current of 20 nA, and a 1-μm defocused beam. Standard RMs used for the analysis included USNM 143965 Kakanui Hornblende (Si, Fe), USNM 122142 Kakanui Augite (Mg), USNM 111356 Arenal Hornblende (Ca), Astimex Rutile (Ti), Astimex Almandine (Al), Astimex Rhodonite (Mn), Astimex Albite (Na), Astimex Sanidine (K), Astimex Tugtupite (Cl), and Astimex Fluorite (F). Peak/background counting times and detection limits (in oxide wt %) for the elements are as follows: Si = 20/10 s, 0.099(8) wt %; Ti = 30/15 s, 0.029(1) wt %; Al = 30/15 s, 0.054(8) wt %; Cr = 60/30 s, 0.021(8) wt %; Fe = 20/10 s, 0.034(6) wt %; Mn = 30/15 s, 0.039(4) wt %; Mg = 20/10 s, 0.029(2) wt %; Ca = 30/15 s, 0.022(6) wt %; Na = 20/10 s, 0.074(9) wt %; K = 30/15 s, 0.016(9) wt %; Cl = 30/15 s, 0.023(2) wt %; and Si = 30/15 s, 0.020(4) wt %. Calculations of the physical-chemical conditions of pressure and temperature amphibole crystallization were carried out using the method of (*103*, *104*) and (*105*).

## Supplementary Materials

### The PDF file includes:

Legends for tables S1 to S6 References
