# Supplementary material for: Granitic intrusions enhance strain localization and rapid mantle exhumation along an oceanic detachment fault
Source: Sci Adv. 2026 Jun 3;12(23):eaec6950. doi: 10.1126/sciadv.aec6950 (PMC13232567; doi:10.1126/sciadv.aec6950)
Supplement: Supplementary file 1 — Legends for tables S1 to S6 References [file sciadv.aec6950_sm.pdf]

Science Advances
AAAS

Supplementary Materials for

Granitic intrusions enhance strain localization and rapid mantle exhumation along an oceanic detachment fault

Eirini M. Poulaki *et al.*

Corresponding author: Eirini M. Poulaki, epoulaki@lsu.edu

Sci. Adv. **12**, eaec6950 (2026)
DOI: 10.1126/sciadv.aec6950

The PDF file includes:

Legends for tables S1 to S6
References

Other Supplementary Material for this manuscript includes the following:

Tables S1 to S6

Supplementary materials include 6 supplementary data tables.

Table 1: Sample names, description and recovery depths

Table 2: Zircon Split-Stream UPb and TE analyses

Table 3: Apatite Split-Stream UPb and TE analyses

Table 4: Stable isotope analyses

Table 5: Amphibole EPMA analyses

Table 6: Ti in quartz analyses

REFERENCES

1. W. R. Buck, L. L. Lavier, A. N. Poliakov, Modes of faulting at mid-ocean ridges. *Nature* **434**, 719–723 (2005).

2. M. Pérez-Gussinyé, J. S. Collier, J. J. Armitage, J. R. Hopper, Z. Sun, C. R. Ranero, Towards a process-based understanding of rifted continental margins. *Nat. Rev. Earth Environ.* **4**, 166–184 (2023).

3. G. S. Lister, G. Banga, A. Feenstra, Metamorphic core complexes of Cordilleran type in the Cyclades, Aegean Sea, Greece. *Geology* **12**, 221–225 (1984).

4. J. A. Olive, M. D. Behn, B. E. Tucholke, The structure of oceanic core complexes controlled by the depth distribution of magma emplacement. *Nat. Geosci.* **3**, 491–495 (2010).

5. M. Cannat, D. Bideau, H. Bougault, Serpentinized peridotites and gabbros in the Mid-Atlantic Ridge axial valley at 15 37′ N and 16 52′ N. *Earth Planet. Sci. Lett.* **109**, 87–106 (1992).

6. L. L. Lavier, W. R. Buck, A. N. Poliakov, Self-consistent rolling-hinge model for the evolution of large-offset low-angle normal faults. *Geology* **27**, 1127–1130 (1999).

7. M. Pérez-Gussinyé, T. J. Reston, Rheological evolution during extension at nonvolcanic rifted margins: Onset of serpentinization and development of detachments leading to continental breakup. *J. Geophys. Res. Solid Earth* **106**, 3961–3975 (2001).

8. M. Bickert, L. Lavier, M. Cannat, How do detachment faults form at ultraslow mid-ocean ridges in a thick axial lithosphere? *Earth Planet. Sci. Lett.* **533**, 116048 (2020).

9. R. L. Gardner, S. Piazolo, N. R. Daczko, P. Trimby, Microstructures reveal multistage melt present strain localisation in mid-ocean gabbros. *Lithos* **366-367**, 105572 (2020).

10. R. L. Gardner, N. R. Daczko, S. Piazolo, The critical role of deformation-assisted melt migration in the formation of oceanic core complexes. *Aust. J. Earth Sci.* **71**, 1–21 (2024).

11. N. J. Montiel, E. Masini, L. Lavier, O. Müntener, S. Calassou, Mantle deformation processes during the rift-to-drift transition at magma-poor margins. *Geochem. Geophys. Geosyst.* **24**, e2023GC010924 (2023).

12. L. Mezri, J. García-Pintado, M. Pérez-Gussinyé, Z. Liu, W. Bach, M. Cannat, Tectonic controls on melt production and crustal architecture during magma-poor seafloor spreading. *Earth Planet. Sci. Lett.* **628**, 118569 (2024).

13. B. Ildefonse, D. K. Blackman, B. E. John, Y. Ohara, D. J. Miller, C. J. MacLeod, Oceanic core complexes and crustal accretion at slow-spreading ridges. *Geology* **35**, 623–626 (2007).

14. C. B. Grimes, B. E. John, M. J. Cheadle, J. L. Wooden, Protracted construction of gabbroic crust at a slow spreading ridge: Constraints from $^{206}Pb/^{238}U$ zircon ages from Atlantis Massif and IODP Hole U1309D (30°N, MAR). *Geochem. Geophys. Geosyst.* **9**, Q08012 (2008).

15. M. Cannat, C. Mével, D. Stakes, "Normal ductile shear zones at an oceanic spreading ridge: Tectonic evolution of Site 735 gabbros (southwest Indian Ocean)," in *Proceedings of the Ocean Drilling Program, Scientific Results* (Ocean Drilling Program, 1991), vol. 118, pp. 415–429).

16. J. Lissenberg, C. J. MacLeod, A reactive porous flow control on mid-ocean ridge magmatic evolution. *J. Petrol.* **57**, 2195–2220 (2016).

17. C. Ferrando, M. Godard, B. Ildefonse, E. Rampone, Melt transport and mantle assimilation at Atlantis Massif (IODP Site U1309): Constraints from geochemical modeling. *Lithos* **323**, 24–43 (2018).

18. E. Albers, T. Schroeder, W. Bach, Melt impregnation of mantle peridotite facilitates high-temperature hydration and mechanical weakening: Implications for oceanic detachment faults. *Geochem. Geophys. Geosyst.* **20**, 84–108 (2019).

19. W.-Q. Zhang, C.-Z. Liu, H. J. B. Dick, Evidence for multi-stage melt transport in the lower ocean crust: The Atlantis Bank Gabbroic Massif (IODP Hole U1473A, SW Indian Ridge). *J. Petrol.* **61**, egaa082 (2020).

20. M.-A. Kaczmarek, A. Tommasi, Anatomy of an extensional shear zone in the mantle, Lanzo massif, Italy. *Geochem. Geophys. Geosyst.* **12**, Q0AG06 (2011).

21. S. Picazo, M. Cannat, A. Delacour, J. Escartín, S. Rouméjon, S. Silantyev, Deformation associated with the denudation of mantle-derived rocks at the Mid-Atlantic Ridge 13°–15° N: The role of magmatic injections and hydrothermal alteration. *Geochem. Geophys. Geosyst.* **13**, Q0AF03 (2012).

22. J. Escartin, G. Hirth, B. Evans, Effects of serpentinization on the lithospheric strength and the style of normal faulting at slow-spreading ridges. *Earth Planet. Sci. Lett.* **151**, 181–189 (1997).

23. J. M. Warren, G. Hirth, Grain size sensitive deformation mechanisms in naturally deformed peridotites. *Earth Planet. Sci. Lett.* **248**, 438–450 (2006).

24. M. Bickert, M. Cannat, A. Tommasi, S. Jammes, L. Lavier, Strain localization in the root of detachment faults at a melt-starved mid-ocean ridge: A microstructural study of Abyssal Peridotites from the Southwest Indian Ridge. *Geochem. Geophys. Geosyst.* **22**, e2020GC009434 (2021).

25. J. J. Schwartz, B. E. John, M. J. Cheadle, E. A. Miranda, C. B. Grimes, J. L. Wooden, H. J. Dick, Dating the growth of oceanic crust at a slow-spreading ridge. *Science* **310**, 654–657 (2005).

26. M. Rioux, S. Bowring, P. Kelemen, S. Gordon, F. Dudás, R. Miller, Rapid crustal accretion and magma assimilation in the Oman-UAE ophiolite: High precision U-Pb zircon geochronology of the gabbroic crust. *J. Geophys. Res. Solid Earth* **117**, B07201 (2012).

27. W.-Q. Zhang, C.-Z. Liu, C. J. MacLeod, C. J. Lissenberg, The role of detachment faulting in the genesis of oceanic felsic melts. *Commun. Earth Environ.* **6**, 109 (2025).

28. N. Zitellini, A. Malinverno, E. R. Estes, Expedition 402 Scientists, "Tyrrhenian continent–ocean transition," in *Proceedings of the International Ocean Discovery Program* (International Ocean Discovery Program, 2025), vol. 402.

29. A. Sanfilippo, A. Pandey, N. Akizawa, E. Poulaki, E. Cunningham, M. Bickert, C. Lei, P. Vannucchi, E. R. Estes, A. Malinverno, N. Abe, A. D. Stefano, I. Y. Filina, Q. Fu, S. B. L. Gontharet, L. E. Kearns, R. K. Koorapati, M. F. Loreto, L. Magri, W. Menapace, V. L. Pavlovics, P. A. Pezard, M. A. Rodriguez-Pilco, B. D. Shuck, X. Zhao, C. Garrido, D. Brunelli, T. Morishita, N. Zitellini, Heterogeneous Earth's mantle drilled at an embryonic ocean. *Nat. Commun.* **16**, 2016 (2025).

30. A. Malinverno, W. B. F. Ryan, Extension in the Tyrrhenian Sea and shortening in the Apennines as result of arc migration driven by sinking of the lithosphere. *Tectonics* **5**, 227–245 (1986).

31. C. Faccenna, F. Funiciello, D. Giardini, P. Lucente, Episodic back-arc extension during restricted mantle convection in the Central Mediterranean. *Earth Planet. Sci. Lett.* **187**, 105–116 (2001).

32. M. F. Loreto, N. Zitellini, C. R. Ranero, C. Palmiotto, M. Prada, Extensional tectonics during the Tyrrhenian back-arc basin formation and a new morpho-tectonic map. *Basin Res.* **33**, 138–158 (2021).

33. F. Trincardi, N. Zitellini, The rifting of the Tyrrhenian Basin. *Geo Mar. Lett.* **7**, 1–6 (1987).

34. K. A. Kastens, J. Mascle, "The geological evolution of the Tyrrhenian Sea: An introduction to the scientific results of ODP Leg 107," in *Proceedings of the Ocean Drilling Program, Scientific Results* (Ocean Drilling Program, 1990), vol. 107, pp. 3–26.

35. N. Zitellini, C. R. Ranero, M. F. Loreto, M. Ligi, M. Pastore, F. D'Oriano, V. Sallares, I. Grevemeyer, S. Moeller, M. Prada, Recent inversion of the Tyrrhenian Basin. *Geology* **48**, 123–127 (2020).

36. R. Sartori, "Bedrock geology of the Tyrrhenian Sea: Insight on Alpine paleogeography and magmatic evolution of the basin" in *CROP Project: Deep Seismic Exploration of the Central Mediterranean and Italy*, I. Finetti, Ed. (Elsevier, 2005), pp. 69–80.

37. M. Prada, C. R. Ranero, V. Sallarès, N. Zitellini, I. Grevemeyer, Mantle exhumation and sequence of magmatic events in the Magnaghi–Vavilov Basin (Central Tyrrhenian, Italy): New constraints from geological and geophysical observations. *Tectonophysics* **689**, 133–142 (2016).

38. M. Prada, V. Sallarès, C. R. Ranero, M. G. Vendrell, I. Grevemeyer, N. Zitellini, R. de Franco, Spatial variations of magmatic crustal accretion during the opening of the Tyrrhenian back-arc from wide-angle seismic velocity models and seismic reflection images. *Basin Res.* **30**, 124–141 (2018).

39. E. Bonatti, M. Seyler, J. Channell, J. Girardeau, G. Mascle, "Peridotites drilled from the Tyrrhenian Sea, ODP Leg 107," in *Proceedings of the Ocean Drilling Program, Scientific Results* (Ocean Drilling Program, 1990), vol. 107, pp. 37–47).

40. N. Zitellini, A. Malinverno, E. R. Estes, N. Abe, N. Akizawa, M. Bickert, E. H. Cunningham, A. Di Stefano, I. Y. Filina, Q. Fu, S. Gontharet, L. E. Kearns, R. K. Koorapati, C. Lei, M. F. Loreto, L. Magri, W. Menapace, T. Morishita, A. Pandey, V. L. Pavlovics, P. A. Pezard, E. M. Poulaki, M. A. Rodriguez-Pilco, A. Sanfilippo, B. D. Shuck, P. Vannucchi, X. Zhao, "Site U1614," in *Proceedings of the International Ocean Discovery Program* (International Ocean Discovery Program, 2025).

41. A. Malinverno, N. Zitellini, E. R. Estes, N. Abe, N. Akizawa, M. Bickert, E. H. Cunningham, A. Di Stefano, I. Y. Filina, Q. Fu, S. Gontharet, L. E. Kearns, R. K. Koorapati, C. Lei, M. F. Loreto, L. Magri, W. Menapace, T. Morishita, A. Pandey, V. L. Pavlovics, P. A. Pezard, E. M. Poulaki, M. A. Rodriguez-Pilco, A. Sanfilippo, B. D. Shuck, P. Vannucchi, X. Zhao, "Site U1612," in *Proceedings of the International Ocean Discovery Program* (International Ocean Discovery Program, 2025).

42. Q. Ma, H. J. B. Dick, B. Urann, H. Zhou, Silica-rich vein formation in an evolving stress field, Atlantis Bank oceanic core complex. *Geochem. Geophys. Geosyst.* **21**, e2019GC008795 (2020).

43. J. Précigout, F. Gueydan, C. J. Garrido, N. Cogné, G. Booth-Rea, Deformation and exhumation of the Ronda peridotite (Spain). *Tectonics* **32**, 1011–1025 (2013).

44. J. D. Clemens, G. Stevens, What controls chemical variation in granitic magmas? *Lithos* **134**, 317–329 (2012).

45. J. S. Singleton, S. Mosher, Mylonitization in the lower plate of the Buckskin-Rawhide detachment fault, west-central Arizona: Implications for the geometric evolution of metamorphic core complexes. *J. Struct. Geol.* **39**, 180–198 (2012).

46. M. Stipp, H. Stünitz, R. Heilbronner, S. M. Schmid, Dynamic recrystallization of quartz: Correlation between natural and experimental conditions. *Geol. Soc. Spec. Publ.* **200**, 171–190 (2002).

47. E. A. Belousova, W. L. Griffin, S. Y. O'Reilly, N. L. Fisher, Igneous zircon: Trace element composition as an indicator of source rock type. *Contrib. Mineral. Petrol.* **143**, 602–622 (2002).

48. T. Geisler, R. T. Pidgeon, R. Kurtz, W. V. Bronswijk, H. Schleicher, Experimental hydrothermal alteration of partially metamict zircon. *Am. Mineral.* **88**, 1496–1513 (2003).

49. E. A. Belousova, W. L. Griffin, S. Y. O'Reilly, N. I. Fisher, Apatite as an indicator mineral for mineral exploration: Trace-element compositions and their relationship to host rock type. *J. Geochem. Explor.* **76**, 45–69 (2002).

50. C. Zhang, J. Koepke, M. Albrecht, I. Horn, F. Holtz, Apatite in the dike-gabbro transition zone of mid-ocean ridge: Evidence for brine assimilation by axial melt lens. *Am. Mineral.* **102**, 558–570 (2017).

51. E. Harlov, Apatite: A fingerprint for metasomatic processes. *Elements* **11**, 171–176 (2015).

52. B. Wenner, H. P. Taylor, Oxygen and hydrogen isotope studies of the serpentinization of ultramafic rocks in oceanic environments and continental ophiolite complexes. *Am. J. Sci.* **273**, 207–239 (1973).

53. P. J. Saccocia, J. S. Seewald, W. C. Shanks III, Oxygen and hydrogen isotope fractionation in serpentine–water and talc–water systems from 250 to 450°C, 50 MPa. *Geochim. Cosmochim. Acta* **73**, 6789–6804 (2009).

54. P. Agrinier, G. Cornen, M. O. Beslier, R. B. Whitmarsh, “Mineralogical and oxygen isotopic features of serpentinites recovered from the ocean/continent transition in the Iberia Abyssal Plain,” in *Proceedings of the Ocean Drilling Program, Scientific Results* (National Science Foundation, 1996), pp. 541–552.

55. D. Skelton, J. W. Valley, The relative timing of serpentinisation and mantle exhumation at the ocean–continent transition, Iberia: Constraints from oxygen isotopes. *Earth Planet. Sci. Lett.* **178**, 327–338 (2000).

56. G. Hirth, D. Kohlstedt, Rheology of the upper mantle and the mantle wedge: A view from the experimentalists. *Geophys. Monogr. Ser.* **138**, 83–105 (2003).

57. J. Ruh, W. Behr, L. Tokle, Effect of grain-size and textural weakening in polyphase crustal and mantle lithospheric shear zones. *Tektonika* **2**, 91–110 (2024).

58. M. Bickert, M.-A. Kaczmarek, D. Brunelli, M. Maia, T. F. C. Campos, S. E. Sichel, Fluid-assisted grain size reduction leads to strain localization in oceanic transform faults. *Nat. Commun.* **14**, 4087 (2023).

59. L. Casini, M. Maino, A. Sanfilippo, B. Ildefonse, H. J. B. Dick, High-temperature strain localization and the nucleation of Oceanic Core Complexes (16.5°N, Mid-Atlantic Ridge). *J. Geophys. Res. Solid Earth* **126**, e2021JB022215 (2021).

60. L. Mehl, G. Hirth, Plagioclase preferred orientation in layered mylonites: Evaluation of flow laws for the lower crust. *J. Geophys. Res. Solid Earth* **113**, B05202 (2008).

61. J. Escartín, C. Mével, S. Petersen, D. Bonnemains, M. Cannat, M. Andreani, N. Augustin, A. Bezos, V. Chavagnac, Y. Choi, M. Godard, K. Haaga, C. Hamelin, B. Ildefonse, J. Jamieson, B. John, T. Leleu, C. J. MacLeod, M. Massot-Campos, P. Nomikou, J. A. Olive, M. Paquet, C. Rommevaux, M. Rothenbeck, A. Steinfuhrer, M. Tominaga, L. Triebe, R. Campos, N. Gracias, R. Garcia, Tectonic structure, evolution, and the nature of oceanic core complexes and their detachment fault zones (13°20′N and 13°30′N, Mid-Atlantic Ridge). *Geochem. Geophys. Geosyst.* **18**, 1451–1482 (2017).

62. I. Raffi, A. Notaro, Reassessment of the age of basement in the Vavilov Basin drilled at ODP Site 651 (Tyrrhenian Sea, Mediterranean). *Riv. Ital. Paleontol. Stratigr.* **131**, 1–10 (2025).

63. B. J. deMartin, R. A. Sohn, J. Pablo Canales, S. E. Humphris, Kinematics and geometry of active detachment faulting beneath the Trans-Atlantic Geotraverse (TAG) hydrothermal field on the Mid-Atlantic Ridge. *Geology* **35**, 711–714 (2007).

64. R. Parnell-Turner, R. A. Sohn, C. Peirce, T. J. Reston, C. J. MacLeod, R. C. Searle, N. M. Simão, Oceanic detachment faults generate compression in extension. *Geology* **45**, 923–926 (2017).

65. H. J. B. Dick, C. J. MacLeod, P. Blum, N. Abe, D. K. Blackman, J. A. Bowles, M. J. Cheadle, K. Cho, J. Ciazela, J. R. Deans, V. P. Edgcomb, C. Ferrando, L. France, B. Ghosh, B. Ildefonse, B. John, M. A. Kendrick, J. Koepke, J. A. M. Leong, C. Liu, Q. Ma, T. Morishita, A. Morris, J. H. Natland, T. Nozaka, O. Pluemper, A. Sanfilippo, J. B. Sylvan, M. A. Tivey, R. Tribuzio, G. Viegas, Dynamic accretion beneath a slow-spreading ridge segment: IODP Hole 1473A and the Atlantis Bank Oceanic Core Complex. *J. Geophys. Res. Solid Earth* **124**, 12631–12659 (2019).

66. G. Baines, M. J. Cheadle, B. E. John, J. J. Schwartz, The rate of oceanic detachment faulting at Atlantis Bank, SW Indian Ridge. *Earth Planet. Sci. Lett.* **273**, 105–114 (2008).

67. M. Allard, B. Ildefonse, É. Oliot, F. Barou, Plastic deformation of plagioclase in oceanic gabbro accreted at a slow-spreading ridge (Hole U1473A, Atlantis Bank, Southwest Indian Ridge). *J. Geophys. Res. Solid Earth* **126**, e2021JB021964 (2021).

68. R. Frost, J. S. Beard, On silica activity and serpentinization. *J. Petrol.* **48**, 1351–1368 (2007).

69. W. Bach, F. Klein, The petrology of seafloor rodingites: Insights from geochemical reaction path modeling. *Lithos* **112**, 103–117 (2009).

70. V. Trommsdorff, B. W. Evans, Alpine metamorphism of peridotitic rocks. *Schweiz. Mineral. Petrogr. Mitt.* **54**, 333–352 (1974).

71. M. Clément, J. A. Padrón-Navarta, A. Tommasi, Interplay between fluid extraction mechanisms and antigorite dehydration reactions (Val Malenco, Italian Alps). *J. Petrol.* **60**, 1935–1962 (2019).

72. G. Hirth, C. Teyssier, J. W. Dunlap, An evaluation of quartzite flow laws based on comparisons between experimentally and naturally deformed rocks. *Int. J. Earth Sci.* **90**, 77–87 (2001).

73. G. Hirth, S. Guillot, Rheology and tectonic significance of serpentinite. *Elements* **9**, 107–113 (2013).

74. F. Bachmann, R. Hielscher, H. Schaeben, Texture analysis with MTEX—Free and open source software toolbox. *Solid State Phenom.* **160**, 63–68 (2010).

75. P. Skemer, I. Katayama, Z. Jiang, S. I. Karato, The misorientation index: Development of a new method for calculating the strength of lattice-preferred orientation. *Tectonophysics* **411**, 157–167 (2005).

76. N. J. Hunter, R. F. Weinberg, C. J. Wilson, V. Luzin, S. Misra, Microscopic anatomy of a "hot-on-cold" shear zone: Insights from quartzites of the Main Central Thrust in the Alaknanda region (Garhwal Himalaya). *Geol. Soc. Am. Bull.* **130**, 1519–1539 (2018).

77. M. Faleiros, R. D. Moraes, M. Pavan, G. A. D. C. Campanha, A new empirical calibration of the quartz c-axis fabric opening-angle deformation thermometer. *Tectonophysics* **671**, 173–182 (2016).

78. M. Wiedenbeck, P. Allé, F. Corfu, W. L. Griffin, M. Meier, F. Oberli, A. von Quadt, J. C. Roddick, W. Spiegel, Three natural zircon standards for U-Th-Pb, Lu-Hf, trace element and REE analyses. *Geostand. Newsl.* **19**, 1–23 (1995).

79. S. E. Jackson, N. J. Pearson, W. L. Griffin, E. A. Belousova, The application of laser ablation-inductively coupled plasma-mass spectrometry to in situ U–Pb zircon geochronology. *Chem. Geol.* **211**, 47–69 (2004).

80. R. C. Kylander-Clark, B. R. Hacker, J. M. Cottle, Laser-ablation split-stream ICP petrochronology. *Chem. Geol.* **345**, 99–112 (2013).

81. J. Sláma, J. Košler, D. J. Condon, J. L. Crowley, A. Gerdes, J. M. Hanchar, M. S. A. Horstwood, G. A. Morris, L. Nasdala, N. Norberg, U. Schaltegger, B. Schoene, M. N. Tubrett, M. J. Whitehouse, Plešovice zircon—A new natural reference material for U–Pb and Hf isotopic microanalysis. *Chem. Geol.* **249**, 1–35 (2008).

82. W. R. Dickinson, G. E. Gehrels, U–Pb ages of detrital zircons from Permian and Jurassic eolian sandstones of the Colorado Plateau, USA: Paleogeographic implications. *Sediment. Geol.* **163**, 29–66 (2003).

83. K. P. Jochum, U. Weis, B. Stoll, D. Kuzmin, Q. Yang, I. Raczek, D. E. Jacob, A. Stracke, K. Birbaum, D. A. Frick, D. Günther, J. Enzweiler, Determination of reference values for NIST SRM 610–617 glasses following ISO guidelines. *Geostand. Geoanal. Res.* **35**, 397–429 (2011).

84. C. Paton, J. Hellstrom, B. Paul, J. Woodhead, J. Hergt, Iolite: Freeware for the visualisation and processing of mass spectrometric data. *J. Anal. At. Spectrom* **26**, 2508–2518 (2011).

85. M. Ferry, E. B. Watson, New thermodynamic models and revised calibrations for the Ti-in-zircon and Zr-in-rutile thermometers. *Contrib. Mineral. Petrol.* **154**, 429–437 (2007).

86. J. M. Ferry, E. B. Watson, New thermodynamic models and revised calibrations for the Ti-in-zircon and Zr-in-rutile thermometers. *Contrib Mineral Petrol* **154**, 429–437 (2007).

87. P. Vermeesch, IsoplotR: A free and open toolbox for geochronology. *Geosci. Front.* **9**, 1479–1493 (2018).

88. F. E. Apen, C. J. Wall, J. M. Cottle, M. D. Schmitz, A. R. C. Kylander-Clark, G. G. E. Seward, Apatites for destruction: Reference apatites from Morocco and Brazil for U-Pb petrochronology and Nd and Sr isotope geochemistry. *Chem. Geol.* **590**, 120689 (2022).

89. F. W. McDowell, W. C. McIntosh, K. A. Farley, A precise ^{40}Ar–^{39}Ar reference age for the Durango apatite (U–Th)/He and fission-track dating standard. *Chem. Geol.* **214**, 249–263 (2005).

90. I. M. Coulson, M. E. Villeneuve, G. M. Dipple, R. A. Duncan, J. K. Russell, J. K. Mortensen, Time-scales of assembly and thermal history of a composite felsic pluton: Constraints from the Emerald Lake area, northern Canadian Cordillera, Yukon. *J. Volcanol. Geotherm. Res.* **114**, 331–356 (2002).

91. D. M. Chew, P. J. Sylvester, M. N. Tubrett, U–Pb and Th–Pb dating of apatite by LA-ICPMS. *Chem. Geol.* **280**, 200–216 (2011).

92. S. N. Thomson, G. E. Gehrels, J. Ruiz, R. Buchwaldt, Routine low-damage apatite U-Pb dating using laser ablation–multicollector–ICPMS. *Geochem. Geophys. Geosyst.* **13**, Q0AF09 (2012).

93. S. Stacey, J. D. Kramers, Approximation of terrestrial lead isotope evolution by a two-stage model. *Earth Planet. Sci. Lett.* **26**, 207–221 (1975).

94. O. K. Droubi, A. M. Bauer, C. Bonamici, W. O. Nachlas, M. J. Tappa, J. M. Garber, J. R. Reimink, U-Th-Pb and trace element evaluation of existing titanite and apatite LA-ICP-MS reference materials and determination of ^{208}Pb/^{232}Th-^{206}Pb/^{238}U date discordance in archaean accessory phases. *Geostand. Geoanal. Res.* **47**, 337–369 (2023).

95. M. L. Odlum, D. F. Stockli, Geochronologic constraints on deformation and metasomatism along an exhumed mylonitic shear zone using apatite U-Pb, geochemistry, and microtextural analysis. *Earth Planet. Sci. Lett.* **538**, 116177 (2020).

96. Z. D. Sharp, A laser-based microanalytical method for the in situ determination of oxygen isotope ratios of silicates and oxides. *Geochim. Cosmochim. Acta* **54**, 1353–1357 (1990).

97. Z. D. Sharp, V. Atudorei, T. Durakiewicz, A rapid method for determination of hydrogen and oxygen isotope ratios from water and hydrous minerals. *Chem. Geol.* **178**, 197–210 (2001).

98. W. O. Nachlas, G. Hirth, Experimental constraints on the role of dynamic recrystallization on resetting the Ti-in-quartz thermobarometer. *J. Geophys. Res. Solid Earth* **120**, 8120–8137 (2015).

99. B. Thomas, E. B. Watson, F. S. Spear, P. T. Shemella, S. K. Nayak, A. Lanzirotti, TitaniQ under pressure: The effect of pressure and temperature on the solubility of Ti in quartz. *Contrib. Mineral. Petrol.* **160**, 743–759 (2010).

100. S. Kidder, J.-P. Avouac, Y.-C. Chan, Application of titanium-in-quartz thermobarometry to greenschist facies veins and recrystallized quartzites in the Hsüehshan range, Taiwan. *Solid Earth* **4**, 1–21 (2013).

101. J. Kohn, C. J. Northrup, Taking mylonites' temperatures. *Geology* **37**, 47–50 (2009).

102. J. Donovan, H. A. Lowers, B. G. Rusk, Improved electron probe microanalysis of trace elements in quartz. *Am. Mineral.* **96**, 274–282 (2011).

103. F. Ridolfi, A. Renzulli, M. Puerini, Stability and chemical equilibrium of amphibole in calc-alkaline magmas: An overview, new thermobarometric formulations and application to subduction-related volcanoes. *Contrib. Mineral. Petrol.* **160**, 45–66 (2010).

104. E. J. F. Mutch, J. D. Blundy, B. C. Tattitch, F. J. Cooper, R. A. Brooker, An experimental study of amphibole stability in low-pressure granitic magmas and a revised Al-in-hornblende geobarometer. *Contrib. Mineral. Petrol.* **171**, 85 (2016).

105. Y. Liao, C. Wei, H. U. Rehman, Titanium in calcium amphibole: Behavior and thermometry. *Am. Mineral.* **106**, 180–191 (2021).

106. E. H. G. Cooperdock, N. H. Raia, J. D. Barnes, D. F. Stockli, E. M. Schwarzenbach, Tectonic origin of serpentinites on Syros, Greece: Geochemical signatures of abyssal origin preserved in a HP/LT subduction complex. *Lithos* **296-299**, 352–364 (2018).

107. S. M. Savin, M. Lee, Isotopic studies of phyllosilicates. *Rev. Mineral. Geochem.* **19**, 189–223 (1988).

108. D. B. Wenner, H. P. Taylor Jr., Temperatures of serpentinization of ultramafic rocks based on O18/O16 fractionation between coexisting serpentine and magnetite. *Contrib. Mineral. Petrol.* **32**, 165–185 (1971).

109. Y. F. Zheng, Calculation of oxygen isotope fractionation in hydroxyl-bearing silicates. *Earth Planet. Sci. Lett.* **120**, 247–263 (1993).

110. W. O. Nachlas, D. L. Whitney, C. Teyssier, B. Bagley, A. Mulch, Titanium concentration in quartz as a record of multiple deformation mechanisms in an extensional shear zone. *Geochem. Geophys. Geosyst.* **15**, 1374–1397 (2014).

111. E. D. Ghent, M. Z. Stout, TiO2 activity in metamorphosed pelitic and basic rocks: principles and applications to metamorphism in southeastern Canadian Cordillera. *Contrib. Mineral. Petrol.* **86**, 248–255 (1984).

112. T. Kawasaki, Y. Osanai, Empirical thermometer of TiO_2 in quartz for ultrahigh-temperature granulites of East Antarctica. *Geol. Soc. London Spec. Publ.* **308**, 419–430 (2008).

113. L. C. Storm, F. S. Spear, Application of the titanium-in-quartz thermometer to pelitic migmatites from the Adirondack Highlands, New York. *J. Metam. Geol.* **27**, 479–494 (2009).

114. K. T. Ashley, L. E. Webb, F. S. Spear, J. B. Thomas, *P-T-D* histories from quartz: A case study of the application of the TitaniQ thermobarometer to progressive fabric development in metapelites. *Geochem. Geophys. Geosyst.* **14**, 3821–3843 (2013).

115. M. Haertel, M. Herwegh, T. Pettke, Titanium-in-quartz thermometry on synkinematic quartz veins in a retrograde crustal-scale normal fault zone. *Tectonophysics* **608**, 468–481 (2013).

116. E. M. Peterman, M. Grove, Growth conditions of symplectic muscovite+ quartz: Implications for quantifying retrograde metamorphism in exhumed magmatic arcs. *Geology* **38**, 1071–1074 (2010).

117. L. Menegon, P. Nasipuri, H. Stünitz, H. Behrens, E. Ravna, Dry and strong quartz during deformation of the lower crust in the presence of melt. *J. Geophys. Res. Solid Earth* **116**, B10410 (2011).

118. F. S. Spear, K. T. Ashley, L. E. Webb, J. B. Thomas, Ti diffusion in quartz inclusions: implications for metamorphic time scales. *Contrib. Mineral. Petrol.* **164**, 977–986 (2012).

119. W. M. Behr, J. P. Platt, A naturally constrained stress profile through the middle crust in an extensional terrane. *Earth Planet. Sci. Lett.* **303**, 181–192 (2011).
